# Breaking the Silence: Critical discussion of a youth participatory action research project

**DOI:** 10.1002/jcv2.12283

**Published:** 2024-11-07

**Authors:** Jaspar Khawaja, Christopher Bagley, Becky Taylor

**Affiliations:** ^1^ University College London London UK; ^2^ States of Mind Social Enterprise London UK

**Keywords:** critical psychology, educational psychology, student voice, youth participatory action research

## Abstract

**Background:**

This paper aims to inform practice for educational psychologists and other professionals who seek to facilitate youth participatory action research (YPAR) in schools. Youth participatory action research is founded on the assumption that young people are capable of being researchers who can co‐create knowledge and act to change the world. It is a worldview as well as a research approach and can be initiated to co‐produce knowledge, facilitate critical thinking, promote the evaluation of social systems and/or act against social oppression.

**Methods:**

We (a) outline the origins of YPAR and review crucial methodological elements of YPAR found in the literature, (b) support practitioners to use a YPAR approach in UK schools using a real‐world example to apply theory to practice and (c) critically discuss outcomes and challenges of facilitating YPAR. An ongoing YPAR project, Breaking the Silence (BtS), facilitated by social enterprise States of Mind and IOE, UCL's Faculty of Education and Society will be outlined within the paper.

**Results:**

BtS demonstrates the power of YPAR to promote the voices of young people, and to allow them to democratically develop action plans that challenge existing education structures. Youth researchers have presented their findings at several conferences and through a national newspaper. They continue to work alongside trade unions and other organisations to push for educational reform. However, the project has demonstrated several challenges and risks of facilitating YPAR. For example, facilitators were not always aware when youth researchers felt they had less meaningful involvement. Facilitators also found it challenging to collaboratively analyse data.

**Conclusions:**

The project indicates that YPAR has the potential to be a democratic, empowering approach that can be brought more widely into the field of education. However, careful considerations are needed by facilitators to mitigate the challenges of the process.

**Key points**

- YPAR is under‐researched and underused in UK schools. This is the first known account of UK EPs facilitating YPAR in academic literature.
- The BtS project provides an example of YPAR being used in practice, which could be replicated and adapted by others.
- YPAR can be an empowering process for young people and lead to them viewing themselves as agents of change.
- The paper outlines considerations that facilitators should take when planning and engaging in projects to mitigate risks, such as exercising power over young people.
- YPAR and similar approaches must have greater value in research and policy if we are to make meaningful change for marginalised groups, by valuing their own experiences, perspectives, priorities, and concerns.

## INTRODUCTION

The current paper aims to inform practice for educational psychologists (EPs) and other professionals who seek to facilitate youth participatory action research (YPAR) in schools and to support practitioners choosing to adopt this approach. Youth participatory action research is under‐researched and underused in UK schools, and this is the first known account of UK EPs facilitating YPAR in academic literature.

Youth participatory action research is an emancipatory approach based on the belief that children and young people (CYP) can, and should, participate as researchers in an inquiry‐based process designed to analyse and act against oppression (Buttimer, 2018b). Youth participatory action research seeks to link reflection (research and analysis) with practice (action), in what Freire (1970) refers to as ‘praxis’. It is an approach that allows for a fluid, flexible and non‐prescriptive methodology that varies based on the needs of participants and their contexts (Cammarota & Fine, 2008).

In this article we critically review the YPAR literature in relation to its origins, epistemological principles and implementation. We then illustrate and elucidate the implementation of YPAR principles using an example. The *Breaking the Silence* (BtS; States of Mind, 2023) project is an ongoing YPAR endeavour conducted across five London secondary schools, by the social enterprise States of Mind,^2^ in collaboration with an Educational Psychology (EP) doctoral student at IOE, UCL's Faculty of Education and Society. The paper includes a discussion of the outcomes, challenges and implications of YPAR, both in relation to previous literature and BtS specifically.

## LITERATURE REVIEW: YOUTH PARTICIPATORY ACTION RESEARCH

A narrative review of literature was chosen following a search focussed on literature published within the last 20 years, using British Education Index, Educational Resources Information Centre and Google Scholar. This was due to the breadth of literature on YPAR across various disciplines and because there are no accounts of YPAR being facilitated by EPs in academic literature. The components of YPAR outlined by Buttimer (2018a) has been used to structure the analysis of literature, as they similarly explored teachers' ability to conduct YPAR, and papers were chosen based on their relevance to EPs working in a school context.

### Origins and terms

Youth participatory action research, a form of PAR, has origins from both the global north and south (Schneider, 2012). Kurt Lewin is one of the founding voices of PAR in the global north and is generally credited with devising the term ‘action research’ (Greenwood & Levin, 2007). Lewin described PAR as a practical approach to solving problems in a cyclical manner of planning, action and reflecting within a democratic climate (Schneider, 2012). The origins of PAR from the global south are highly influenced by the critical pedagogy of Freire (1970), a belief that teaching practices should empower CYP to examine existing inequalities and power imbalances and envision a better world.

### Epistemological principles

Six widely agreed epistemological principles of YPAR are repeated in the literature (Buttimer, 2018a; Cammarota & Fine, 2008; Kirshner, 2010). Firstly, YPAR is *critical in nature*. An essential component is the active critique of systems of power and oppression, alongside taking action to promote change (Buttimer, 2018b). This differs from traditional epistemological stances that value notions of objectivity and neutrality.

Second, YPAR *takes an inquiry stance*. This allows knowledge to be co‐produced between adults and youth researchers to address complex socio‐political questions (Schneider, 2012) and contrasts with typical schooling which involves a set curriculum and content to be ‘learned’ in preparation for standardised tests (Rodríguez & Brown, 2009).

Third, YPAR is *situated in the lived experience of CYP* and fourth, it *draws upon their unique knowledge and expertise*. In general, young people are directly affected by the issues or topics explored as part of YPAR, thus avoiding an ‘intellectual void’ whereby young people's voices are excluded from research and policy decisions that impact their lives (Cammarota, 2008). Youth participatory action research validates the knowledge of local communities as well as their right to co‐produce knowledge and decide what constitutes ‘truth’ (Rodríguez & Brown, 2009).

The fifth principle is the *robust participation of CYP* at each stage of the research. This is to avoid ‘listening’ to CYP in a tokenistic manner and interpreting their views in their absence (Kellet, 2010). Gal's (2017) ecological model of participation demonstrates the range of contextual factors that impact upon children's participation; such as their level of involvement (from being consulted by adults to being decision‐makers), the time they participate (from a one‐off event to continuous involvement), and the extent to which structures encourage or require child participation.

The sixth epistemological principle of YPAR is that the research must be *designed to raise awareness about issues of injustice and create social change*. Actions taken are grounded in knowledge generated and are initiated in response to the emerging findings. Children and young people may also present their work more widely to attempt to maximise its impact (Buttimer, 2018a). Stringer (2007) argues that the best way to measure the success of a YPAR project is by evaluating the social change it generates.

The epistemological principles show how YPAR differs significantly from many ‘traditional approaches’ to research, both in terms of the roles of researchers and participants and the research process. A core criticism of YPAR is that the research is biased, the youth researchers are largely untrained, and the methods used lack rigour (Duncan‐Andrade & Morrell, 2008). This perspective means that YPAR is not prevalent in leading journals and is rarely cited in conversations about educational policy (Duncan‐Andrade & Morrell, 2008). However, the counterargument is that for marginalised groups, meaningful change can only come if they have full, active participation in the research process and have opportunities to produce and use knowledge based on their own experiences, perspectives, priorities and concerns (Duncan‐Andrade & Morrell, 2008). It is a move from extractive research, in which small levels of participation benefit the researcher and the status quo, to co‐produced research attempting to change the lives of people and the world (Kagan, 2012).

### Implementation of youth participatory action research epistemology

Youth participatory action research is an approach that allows for a fluid, flexible and non‐prescriptive methodology that will vary based on the needs of participants and their contexts (Buttimer, 2018a; Cammarota & Fine, 2008). However, some themes emerge from the academic literature on how adult facilitators can engage and work alongside CYP. Broadly speaking, YPAR involves four stages: (a) problem identification, (b) data generation, (c) data analysis, and (d) action (Kornbluh et al., 2015). The stages are not linear and often interact and overlap during the process, and can be repeated as part of a cycle to create progressive change over time (Pine, 2009).

#### Problem identification

The process often starts with youth researchers choosing a research topic that addresses a problem that affects their community (Buttimer, 2018a). Adult researchers may guide the choice of topic through activities, either beforehand or during this process, to ensure that the research is critical and grounded in the lives of CYP (Cammarota, 2016; Kirshner, 2015; Raygoza, 2016).

#### Data generation

For youth researchers to collect data, adult researchers must teach them the skills necessary to do so, including ethics protocols, research design, data collection and analysis approaches, and forms of knowledge dissemination (Kirshner, 2008; Wright, 2015). Youth researchers can then develop methodologies and conduct research alongside adult researchers using tools that they believe are most relevant, such as surveys, interviews, focus groups and ethnographies (Cammarota, 2016; Cammarota & Fine, 2008).

#### Data analysis

After data is collected, the youth researchers conduct qualitative and/or quantitative analyses of the data, often in collaboration with adult researchers who have had more comprehensive research training (Kirshner, 2015). The involvement of youth researchers in data analysis has been highlighted as a common challenge of participatory research due to their age, skills and time constraints (Coad & Evans, 2008). Liebenberg et al. (2020) and Neill et al. (2021) provide detailed guidance and examples of involving youth researchers throughout data analysis.

#### Action

The team produce an action plan based on their findings; which has been argued to be the most important part of the research, because of the emancipatory goal (Buttimer, 2018a). The products of the research should be dynamic, interactive and disseminated in conjunction with youth researchers (Tuck et al., 2008). The aim is to maximise the impact of research and promote social change (Stringer, 2007). Next, the literature on outcomes of YPAR will be reviewed.

### Outcomes of youth participatory action research

Drawing on Shamrova and Cummings' (2017) comprehensive review of 45 YPAR studies, we will discuss outcomes of YPAR at three levels: children and youth, organisations, and communities.

#### Children and youth

At an individual level, YPAR has been found to increase social justice awareness and knowledge, or ‘critical consciousness’. Youth participatory action research can enable CYP to be exposed to social justice issues, develop healthy relationships with adults and feel a sense of belonging in their community, which in turn makes them more likely to display prosocial behaviour and become agents of change. ‘Critical consciousness’, a term popularised by Freire (1970), can be understood as having achieved an in‐depth understanding of the topic researched and comprehension of systemic and structural causes of problems. Additionally, several studies cited research skills, teamwork, and enhanced relationships between adults and CYP as positive outcomes achieved through YPAR (Shamrova & Cummings, 2017).

#### Organisations

At the organisational level, CYP have often become more active participants in service delivery and policy because of access to spaces generally exclusive to adults, for example, city council meetings. Following YPAR, some organisations have developed awareness or advocacy campaigns and/or training programmes informed by the perspectives of CYP during YPAR.

#### Communities

Multiple researchers have referred to the respectful intergenerational dialogue that YPAR enabled within communities. Youth participatory action research has also led to the creation of bodies that further enable CYP's voices, such as youth advisory boards within communities (Malone, 2013). Reported outcomes at the level of communities include menu changes for school lunches (Reich et al., 2015), adaptations to sexual education teaching (Soleimanpour et al., 2008) and improvements to community infrastructure (Shamrova & Cummings, 2017).

### Challenges of facilitating youth participatory action research

While there are significant benefits of engaging CYP in research and as co‐researchers, the assumption that participatory research with CYP is automatically effective has been questioned (Fox, 2013; Horgan, 2017). Naker et al. (2007) suggest that more openness and understanding regarding the challenges of CYP participation can produce some of the richest findings from participatory projects to develop this type of research further.

This section will examine three main factors that contribute to challenges regarding YPAR projects: (a) structural factors, (b) facilitator factors, and (c) student factors.

#### Structural factors

Structural challenges are commonly cited by those who attempt to implement YPAR in schools, including convincing schools to create space in the curriculum for YPAR (Cannella, 2009; Ozer et al., 2010) and funding for YPAR (Shamrova & Cummings, 2017). This challenge is exacerbated by a political and educational climate centred around high stakes testing and a standardised curriculum (Kirshner, 2015). Teachers have found it particularly difficult to find space for YPAR in school time (Mirra et al., 2015; Rubin et al., 2017). Therefore, most accounts of YPAR in academic literature have been conducted outside standard curriculum time (Buttimer, 2018a). Many projects have identified a lack of time as the main difficulty when conducting YPAR projects (Ozer et al., 2010).

#### Facilitator factors

Several ethical issues face facilitators of any form of participatory research with CYP, which can become more problematic than typical research (Wallace & Giles, 2019). Ethical issues include: matters of power and control in the research, the use and value of the research, child protection and confidentiality (Smith et al., 2002). There is currently no ethical guidance or framework for participatory research with CYP published by the British Psychological Society (BPS) or any other similar body.

A key ethical issue is the meaningful participation of CYP (Kellett, 2005; Shamrova & Cummings, 2017). The more the adult researcher is involved in the research, the higher the risk of them exercising power over CYP's opinions and misinterpreting their voices (James, 2007). Although many YPAR projects are time‐limited, expanding the stages in which CYP are involved should be prioritised (Shamrova & Cummings, 2017).

Power relationships are particularly difficult to navigate in PAR, as the aim is to reduce and/or eliminate power imbalances between the adult co‐researchers and youth researchers (Jacobs, 2018). Foucault (1977) discusses how power does not only exist between individuals but in the positions they hold, and therefore an adult co‐researcher could be assumed to hold more power than youth researchers. However, power is not necessarily harmful and can be used alongside others in solidarity rather than power over others (Cornwall & Gaventa, 2001; Park, 2001). Spyrou (2011) states that due to the importance of power within YPAR, adult researchers should use a ‘critical, reflexive approach’ in their research diary to constantly address potential power imbalances at every stage of the research.

Beyond ethical issues, a challenge for facilitators, particularly teachers, is having the necessary research training and skills to enable youth researchers to conduct research and identify links between research and action (Buttimer, 2018a). Educational psychologists could be better placed in this regard, developing research method skills and other relevant skills (e.g., creating a safe space for others and managing conflict) through the commonly applied consultation service delivery (Fallon et al., 2010), which are directly applicable to facilitating YPAR.

#### Student factors

Researchers have suggested that CYP are not used to sharing power with adults in the school context and are more familiar with their voices being excluded rather than encouraged (Kohfeldt et al., 2011). Therefore, it can be tough for them to take on the unfamiliar role of a youth researcher, which asks them to lead the learning process. Furthermore, some youth researchers may find some YPAR tasks challenging, lacking critical skills or being resistant to certain tasks (Foster‐Fishman et al., 2010). Transferring power to young adolescents can be complex due to issues around maturity which could lead to behaviours such as putting peers down (Wilson et al., 2007) and messing around (Ozer et al., 2010). Additionally, power dynamics exist within groups of CYP, which can lead to some voices becoming disempowered, marginalised and excluded from research (Horgan, 2017).

## BREAKING THE SILENCE: AN ILLUSTRATIVE EXAMPLE OF YOUTH PARTICIPATORY ACTION RESEARCH

### Background to Breaking the Silence

Breaking the Silence (States of Mind, 2023) was initiated in 2019 by the social enterprise, States of Mind, an organisation led by young people, psychologists and teachers aimed at co‐creating new knowledge and ways forward around education and mental health provision. Each phase of BtS involves a new cohort of young people who are in Year 12 (ages 16–18) and attending sixth‐form colleges in a London borough. Young people apply to participate in the project and commit to taking part in sessions for a defined period. Breaking the Silence has proceeded in four distinct phases, each beginning at the start of an academic year. Cohorts of youth researchers volunteer to take part for one school year and hence, one Phase of the project. At the end of the school year, they pass the project onto the next cohort to take forward.

Breaking the Silence was initiated in response to two problems. First, the views of young people are rarely meaningfully sought as part of education consultations (Lundy, 2007), in contravention of their fundamental human rights (United Nations, 2009) and the core tenets of democracy. Second, abundant data demonstrates that schooling in England is not working for all CYP and in many cases, is psychologically harmful (WHO, 2020; OECD, 2018; Timpson, 2019; Edge Foundation, 2023; Children's Society, 2019). Alongside the anecdotal experience of EPs working across schools and with CYP, it was felt important to actively seek the participation of CYP in framing the questions and devising their own approaches to explore *why* these trends have been observed.

The adult co‐researchers (a qualified EP throughout all phases, and an EP in Training for Phases 2 and 3) tried to ensure that throughout all phases of BtS, projects were designed by youth researchers, positioning participants as experts in their own lives and educational experiences. A more traditional research project, in contrast, might set the parameters and evaluative measures by for example, presenting a survey to students asking, ‘what can your school do to improve mental health?’. While this sort of research may have value, it positions CYP as passive subjects and does not allow them to define the questions that are most applicable to their lives.

### Breaking the Silence methodology

Phase 1 (States of Mind, 2023) took place in 2019 in response to an upcoming government consultation around school accountability and the purpose of Ofsted (the English education and care inspectorate). 80 young people aged 16–18 from three sixth forms/colleges were asked their views around school inspection and accountability in four focus groups led by an adult researcher from States of Mind. Subsequently, a group of seven youth researchers volunteered to analyse the themes generated, alongside States of Mind psychologists. They decided to compose a letter (States of Mind, 2019b) to Amanda Spielman (His Majesty's Chief Inspector at Ofsted) explaining their findings and proposing solutions. They received a response from Ofsted which they thought failed to respond to their concerns or ideas (States of Mind, 2019a).

During Phase 2, a new cohort of nine youth researchers from one sixth‐form college decided to further investigate the themes from Phase 1. Research questions developed by youth researchers included ‘What is the impact of school on the mental health of young people?’ and ‘How do students feel the education system prepares them for the future?’. Youth researchers co‐constructed a two‐part questionnaire and follow‐up focus group questions alongside two adult researchers (States of Mind psychologist and EP in Training) over seven, one‐hour sessions. 247 participants aged 16–18 answered the questionnaire and 14 participants engaged in four online focus groups. Three youth researchers analysed findings alongside an adult researcher and have presented findings at several conferences and to the UK Parliamentary Education Select Committee (Khawaja et al. in preparation).

Phase 3 built on the previous phases. A new cohort of 12 youth researchers volunteered to work together to co‐produce an education evaluation framework: an alternative to Ofsted. Over 29, 90‐min sessions they studied academic research around school evaluation, analysed the Ofsted framework, designed research questions, generated data and chose how to disseminate it, with whom and where. This included five sessions on developing research questions, questionnaire questions and interview/focus group questions, after youth researchers had chosen those methods to generate data. Youth researchers also had time with an adult researcher to practice their delivery, prior to conducting interviews and focus groups. The youth researchers' questionnaire was completed by 160 students (aged 16–18) and 56 teaching staff. Furthermore, youth researchers conducted two focus groups with students, one focus group with teaching staff, two interviews with head teachers and three interviews with ex‐Ofsted inspectors. Phase 3 concluded with youth researchers producing their own education evaluation framework called the ‘Review for Progress and Development’ (RPD) and co‐creating a documentary outlining their experiences of the project (States of Mind, 2023). Khawaja (2022) used Phase 3 to explore how YPAR can be effectively facilitated.

In Phase 4 (States of Mind, 2023), a new cohort of 12 young people spent an academic year refining the RPD. Again, they read numerous academic studies and ran focus groups with students and teachers with the aim of co‐constructing an evaluation framework that allowed meaningful data to be generated across core areas of school experience. Phase 4 included 10 sessions around planning, running and critiquing the effect of focus group questions and approaches. Building upon the work completed in Phase 3 and amending the RPD following new data, a framework emerged involving continuous school self‐evaluation in partnership with local schools. The aim is for schools to trial the RPD and freely adapt the framework to meet their school community and co‐produce the learning environment that attempts to respond flexibly and authentically to CYP.

### Implementation of youth participatory action research epistemology during Breaking the Silence

As outlined earlier, YPAR has six epistemological principles. Breaking the Silence was influenced by the conceptualisation of ‘complexity reduction’ (Biesta, 2009), which offers a critique of most educational research, as beginning from a position of status quo acceptance and an assumption that the underpinning values, practices and ideologies of the school system are sound. By contrast, BtS allowed youth researchers to *explore critically* (principle 1) the systemic, long‐standing operations of the education system and reimagine alternatives.

Taking an *inquiry stance* (principle 2), adult co‐researchers posed open questions to stimulate deep, critical appraisal of the impact of school systems on their lives. For example, ‘What is the purpose of education?’, ‘What impact has the school system had on you?’ Once youth researchers had spent time considering some of these ‘big questions’, they created their own research questions and were leaders of the inquiry from the beginning.

In regard to the third and fourth principles, BtS represents a unique attempt to elicit, authentically, *the lived experience* (principle 3) of young people around education and mental health, by supporting them to develop research that was meaningful to them, and *engage their knowledge and expertise* (principle 4) to imagine alternatives to current systems.

Allowing for *robust participation* (principle 5), youth researchers were engaged in all aspects of the research, from design to dissemination. However, the extent of youth participation varied at different stages of research, as will be discussed later.

Regarding principle 6, *creating social change,* youth‐led action has been involved at every phase of BtS in an attempt to create psychologically healthy school environments that meet the needs of all CYP.

An analysis of how epistemological principles were implemented is discussed later, however, a more detailed analysis can be found in Khawaja's (2022, p. 76) doctoral research.

### Ethical considerations

For Phases 2 and 3 of BtS, ethical approval was granted by UCL Institute of Education Research Ethics Committee and the projects were registered with UCL Data Protection Office. Phases 1 and 4 were not covered as they were solely conducted by States of Mind. However, The BPS codes of ethical research practice were followed during all phases (BPS, 2021). To ensure that the research conducted was ethical, considerations around consent, confidentiality and data handling were considered. In addition, possibilities of power issues and risk of coercion between adult and youth researchers, specific to YPAR, were carefully considered.

## FINDINGS AND DISCUSSION FROM BREAKING THE SILENCE

This section will highlight findings from the BtS project, offering insights into outcomes from the project and the challenges faced. The purpose of this is to better understand whether it is (a) possible for EPs and other professionals to conduct YPAR in schools, and (b) understand the challenges that need to be considered before and during the facilitation of YPAR in schools.

### Outcomes

Again, outcomes from BtS are discussed at three levels: children and youth, organisations, and communities.

#### Children and youth

There were examples of youth researchers gaining ‘critical consciousness’ through YPAR. For example, during Phase 3, youth researchers initially positioned teachers as sources of stress and pressure, but later described how the systemic pressure placed on teachers feeds down to students, locating problems at a structural, rather than individual, level (Khawaja, 2022, p. 115).

Other outcomes observed during BtS Phase 3 included youth researchers experiencing a greater sense of agency and empowerment having through the YPAR process, alongside increased desire to challenge authority and critique systems (Khawaja, 2022, p. 115). Further, after each phase, a core group of two or three youth researchers have opted to continue their involvement with States of Mind. They form a working group who meet monthly, underpinned by a desire to facilitate social and educational transformation, demonstrating a lasting impact on their political engagement.

#### Organisations

Youth researchers in Phase 1 communicated their findings via a letter to Amanda Spielman (Ofsted Chief Inspector) (States of Mind, 2019b). Following Phases 2 and 3, adult researchers supported youth researchers to present their findings to their colleges and through appropriate platforms. More recently (as part of BtS Phase 5) their work will form part of the National Education Unions' ‘Replace Ofsted’ project, alongside contributions from academic researchers and other educational innovators. The youth researchers have been invited to sit on the Advisory Board of a newly formed group, ‘Rethinking Accountability’, who are also challenging Ofsted's practices. Some are members of working groups set up by organisations such as the Fair Education Alliance and the Edge Foundation as a direct consequence of BtS. Youth participatory action research can therefore create the possibility for further collaboration with organisations.

#### Communities

Breaking the Silence scope has been broader than the level of communities, targeting change at a national level. Youth researchers co‐constructed the RPD (States of Mind, 2023) and a documentary, ‘The Framework’ (States of Mind, 2023) in attempt to reach as wide an audience as possible. They have presented at numerous conferences and accepted invitations to present their findings to professionals including: Headteachers participating in Big Education's (Academy Trust) ‘Big Leadership Adventure’, MPs sitting on the Education Select Committee, the ‘Beyond Ofsted’ Inquiry, and The Guardian (Millar, 2022). Hence, YPAR can support young people to make an active contribution to conversations and action around societal change at varied systemic levels. Whilst impossible to quantify, it is possible that BtS has contributed to changing attitudes towards Ofsted and the widespread drive to change it (NEU, 2024).

### Challenges of facilitating youth participatory action research

#### Structural factors

To implement BtS, years of groundwork and relationship‐building were required. States of Mind had built trusting relationships with the participating schools and colleges; this was crucial given the project raised some challenging themes around the school system and, by implication, practices that were operating in these schools. Further, schools and colleges had already allocated space for weekly 90‐min enrichment sessions which allowed Phases 3 and 4 to proceed with few logistical challenges. Hence, in this instance, initial relationship building, pre‐existing curriculum time and educators willing to try something new meant that structural barriers were reduced, a context in which potential facilitators of YPAR are unlikely to find themselves.

#### Facilitator factors

Throughout all phases of BtS, data analysis was found to be a difficult aspect to facilitate, particularly concerning qualitative data. Quantitative data generated from questionnaires could be shared with youth researchers, who were able to independently pick out the key findings and then discuss them as a group. Qualitative data was harder to analyse, particularly due to confidentiality of participants and the time required to conduct a thematic analysis. Hence, flexible approaches were used. For example, during Phase 2, an adult co‐researcher conducted a thematic analysis independently and then checked in with youth researchers to review and discuss the language that could best describe themes. Phase 3 involved the youth researchers more directly. They conducted interviews/focus groups, fed back the key findings to the whole cohort in follow‐up group sessions, thus ensuring the core themes were shared across the team. Breaking the Silence highlighted how rigorous data analysis is challenging in YPAR as adult researchers had to make decisions about the project priorities. The implications from this are that facilitators may need to consider the priorities of YPAR during time‐limited projects, as there may be a difficult trade‐off between the time needed for rigorous thematic analysis, and the time required for ‘action’.

Khawaja (2022, p.87) evaluated the extent to which meaningful participation for youth researchers took place in Phase 3. Whilst they mostly experienced a sense of autonomy and control, there were periods where they did not feel meaningfully involved in decision‐making. Findings indicated that meaningful participation for youth researchers was easier to enable during times of planning and conducting research, and producing action. Meaningful participation was more difficult during periods of reading literature and data analysis. A key finding was that transparency and ongoing discussions around the role of adult and youth researchers are needed to reduce adults unintentionally exerting power over youth. Khawaja (2022, p. 195) produced an infographic to support facilitators of YPAR on important considerations and guidance on decision making during YPAR.

Facilitators found it challenging to balance the value of taking leading roles at times, with the risk of projecting their own views onto youth researchers. Yang (2009) discusses this tension in what they describe as the two fallacies threatening YPAR. The first is ‘the fallacy of idealized democracy’ in which people mistake the student‐led element of YPAR for equal participation. The aim is not to create a situation without a leader, but to distinguish between authority and authoritarianism in knowledge production. This highlights the need for adults to lead at times, however, our findings demonstrated the importance of being transparent about this with youth researchers. The second fallacy, ‘predetermined criticism,’ occurs when YPAR replicates the facilitator's critical worldviews and new ideas are not constructed, raising questions on whether facilitators should attempt to be impartial and objective. We found that determining adult researchers' impact on youth researchers is challenging; thus, facilitators should use reflective journals to support self‐awareness of bias, allowing considered adjustments (p. 137).

#### Student factors

A consistent challenge throughout BtS relates to young peoples' assumption that adults know best and a tendency to refer to adults as ‘sir’ or ‘miss’. It required time, reminders, and the creation of a safe space to allow youth researchers to feel comfortable working alongside adults as co‐researchers who have an equal say. This indicates that time is required for the prevailing social hierarchies experienced in typical schooling to be circumvented when conducting YPAR (Khawaja, 2022, p. 77).

Breaking the Silence project found that working alongside youth researchers in Year 12 worked well. The reasons for this were three‐fold: there was time in the curriculum when they were not in lessons, they were able to consent to the research independently, and we could contact them directly through their school email rather than plan through school staff. This is not to suggest that YPAR cannot be done with CYP of a younger age, but it is likely to add difficulty.

## CONCLUSION

Youth participatory action research is founded on the assumption that young people are capable of being researchers who can co‐create knowledge and act to change the world. It is a worldview as well as a research approach and can be initiated to co‐produce knowledge, facilitate critical thinking, promote the evaluation of social systems and/or act against social oppression in an ethical, authentic manner that positions young people as active participants, not passive subjects. Whilst there are considerations and ethical issues that must be addressed to conduct YPAR effectively, we support the view of Mirra and Rogers (2016): “If we really do believe in the full humanity of young people, that their voices are valid and should be heard in spaces that make decisions about their schooling experiences, then YPAR is not an extracurricular endeavour but an imperative mandate” (p. 153).

## AUTHOR CONTRIBUTIONS

**Jaspar Khawaja**: Conceptualization; Data curation; Formal analysis; Investigation; Methodology; Project administration; Writing ‐ original draft. **Christopher Bagley**: Conceptualization; Supervision; Writing ‐ review & editing. **Becky Taylor**: Supervision; Writing ‐ review & editing.

## CONFLICT OF INTEREST STATEMENT

The authors have declared that they have no competing or potential conflicts of interest.

## ETHICAL CONSIDERATIONS

For Phases 2 and 3 of BtS, ethical approval was granted by UCL Institute of Education Research Ethics Committee and the projects were registered with UCL Data Protection Office. Phases 1 and 4 were not covered as they were solely conducted by States of Mind. However, The BPS codes of ethical research practice were followed during all phases (BPS, 2021). All participants provided written informed consent before entering the research.

## Data Availability

Research data are not shared.

## ENDNOTES

^2^

https://www.statesofmind.org/
